# Research status and hotspots of oncology genetic nursing: a bibliometric analysis

**DOI:** 10.3389/fonc.2025.1523185

**Published:** 2025-12-17

**Authors:** Ya Hu, Cangmei Fu

**Affiliations:** 1 Department of Urology, Sun Yat-sen University Cancer Center, Guangzhou, China; 2 State Key Laboratory of Oncology in Southern China, Guangzhou, China; 3 Guangdong Provincial Clinical Research Center for Cancer, Guangzhou, China; 4 Department of Oncology, The Second Xiangya Hospital, Central South University, Changsha, China

**Keywords:** bibliometric, CiteSpace, genetic nursing, hotspots, oncology

## Abstract

**Background:**

Cancer is a major global challenge in the 21st century, posing significant social, public health, and economic pressures. Not only does it shorten human lifespans, but it also imposes substantial burdens on society and the macroeconomy. Oncology genetics nursing specialty focuses on providing genetic testing, genomic analysis, and related services to patients. Due to the growing demand for cancer risk assessment, widespread genetic testing, and increasing rates of hereditary cancers, the need for genetic counseling has grown exponentially. To our knowledge, no comprehensive bibliometric study has yet been conducted to systematically compile relevant data in this field.

**Objective:**

This study attempts to review existing literature in the field of oncology genetic nursing, highlight current hot topics, and predict future development trends.

**Methods:**

A bibliometric analysis was conducted by searching relevant literature on oncology genetic nursing from January 1, 1999 to August 6th, 2025 in the Web of Science Core Collection (WOSCC). The CiteSpace software was used to map publication volume, country and author collaboration networks, keyword co-occurrences, and word emergence.

**Results:**

The study analyzed 658 articles from January 1, 1999 to August 6th, 2025. The United States leads in cooperative publications in oncology genetic nursing, with 407 of total publications, including the top 10 institutions, most published authors (*N=28*), and most cited authors (*N=47*). Breast cancer (*N=146*), ovarian cancer (*N=40*) and colorectal cancer (*N=25*) are the main diseases studied. Primary research hotspots include offering nurse-led genetic risk assessments, genetic testing, and genetic counseling services, as well as improving the training of senior oncology genetic practice nurses. Emerging trends focus on genetic testing, genetic counseling and precision medicine in oncology genetic nursing.

**Conclusion:**

This bibliometric study maps the research hotspots and trends for the past more than 26 years  in oncology genetic nursing. Our findings will enable researchers to better understand trends in this field and find suitable directions and partners for future research.

## 1 Introduction

Cancer poses formidable societal, public health, and economic challenges in the 21st century. Global Cancer Statistics 2022 revealed a staggering burden: nearly 20 million new cancer cases and 9.7 million cancer-related deaths were recorded that year alone (1). Current estimates suggest that approximately one in five people will develop cancer in their lifetime and that about one in nine men and one in twelve women will die from the disease (1). The World Health Organization (WHO) projects a substantial increase, anticipating approximately 27 million new cases and 17.5 million cancer-related deaths annually by 2050 (2). Beyond impeding gains in life expectancy, cancer imposes significant societal and macroeconomic costs and remains a leading cause of disability and mortality, presenting an escalating challenge to global public health systems (1–3).

Oncology genetic nursing is a specialized discipline dedicated to providing genetic, genomic, and related healthcare services to patients (4, 5). Oncology genetics nursing plays a key role in disease prediction and prevention by helping to collect, evaluate and disseminate information to assist physicians in helping patients make treatment decisions and monitor treatment outcomes and adverse drug reactions (6, 7). Cancer genetic nurses lead genetic services that are cost-effective and effective, and their counselling clinics contribute significantly to primary care. A study by the University of Nottingham Hospital in the United Kingdom (UK) showed that advanced practice nurse-led cancer genetic services significantly reduced patient waiting times (8), provided better and more streamlined services for patients undergoing genetic testing, and enabled oncologists and patients to consider personalized treatment plans at an earlier stage. A study on the feasibility and acceptability of genetic counselling clinics led by genetic nurses in primary care in the United Kingdom (UK) found that patients had high overall satisfaction, were satisfied with the time and distance required to travel to and from the hospital (9), and had low consultation costs. With the sharp increase in demand for cancer genetic counselling and the shortage of genetic services professionals (10, 11). Advanced practice nurses in oncology will play a key role in cancer risk assessment, genetic counselling, genetic testing, management of hereditary cancer families, and psychosocial support (12).

Decades of research have yielded a substantial body of literature on oncology genetic nursing (13, 14). However, the rapidly expanding volume of publications makes it difficult for practitioners and researchers to update their knowledge in a timely manner. While traditional narrative reviews consolidate references and track field development, they rely on subjective interpretation, which can introduce bias and incomplete perspectives (15). In contrast, bibliometrics offers a quantitative approach to evaluating information and objectively identifying research hotspots and evolving trends within a discipline (16). Beyond statistical analysis, bibliometrics enables visualization of knowledge structures through bibliographic mapping techniques (17). CiteSpace is a Java application widely used for scientometrics and data visualization, capable of intuitively revealing research hotspots, emerging frontiers, and evolutionary paths in specific academic fields through the construction of knowledge maps (18). Consequently, to our knowledge, this study presents the first bibliometric review focused specifically on oncology genetic nursing, employing graphical analysis to examine publications, countries, authors, journals, references, and keywords pertinent to this field of research. This study aims to review existing literature in the field of oncology genetic nursing, highlight current hot topics, and predict future development trends.

## 2 Materials and methods

### 2.1 Data collection

**Figure 1:**
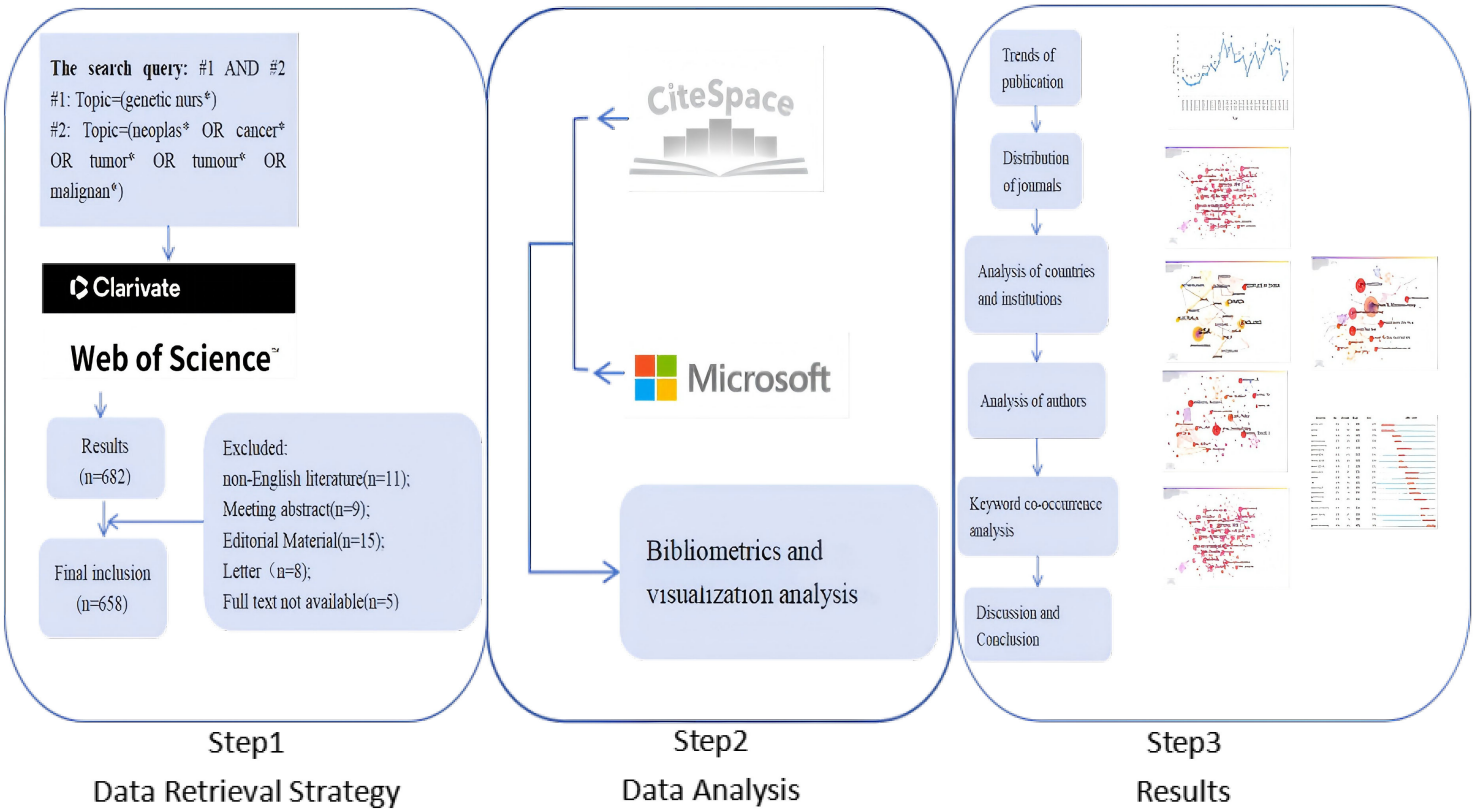
The detailed research process.

The Web of Science Core Collection (WOSCC) is a highquality, up-to-date, error-free database of more than 12,000 of the most influential and valuable scientific journals (17). All relevant references have been searched and updated from the database’s inception in January 1, 1999 until August 6th, 2025, in the Science Citation Index Expanded/SCI-E and Social Sciences Citation Index (SSCI) database in the WOSCC, which is most suitable for bibliometric analysis (19, 20). Using a search strategy agreed upon by all authors, the following criteria were employed: Topic=genetic nurs* AND Topic=(neoplas* OR cancer* OR tumor* OR tumour* OR malignan*). The specific search query is illustrated in **Table 1**. The study covers research from 1999-2025, with 682 papers obtained from the WOSCC SCI-E database, including all clinical and preclinical papers. Inclusion criteria: Published in peer-reviewed academic journals; content pertaining to oncology genetic nursing. Exclusion criteria: Letters, news articles, conference proceedings, and popular science literature. With the literature types being screened, non-English papers (n=11), meeting abstract (n=9), letters (n = 8), editorial material (n=15), and full text not available (n=5) are excluded.The detailed literature screening process is shown in **Figure 1**. Finally, a total of 658 publications are included in the final bibliometric and visual analysis. In conclusion, the relevant data in the form of plain text format from WOSCC are exported, including title, author, year of publication, country, institution, keywords, citations, abstracts, and references.

**Table 1:** Search strategy.

Search platform	Sources searched	Time span	Search query	Document types	Language restriction
WOSCC	SCI-E, SSCI	From 1999-01-01 to 2025-08-06	Topic: (“genetic nurs*”) AND Topic: (“neoplas*” OR “cancer*” OR “tumor*” OR “tumour*” OR “malignan*”)	All types initially retrieved	All languages initially retrieved

### 2.2 Data analysis and visualization

The study group adopted Microsoft Excel 2020 to draw the annual publication graph and a chart of the top 10 countries in terms of the number of publications. The group also used bibliometrics analysis software (CiteSpace 6.1. R6 Basic) to analyze and visualize the data of the above 658 documents.

CiteSpace is a visualization software developed by Professor Chaomei Chen that can explore hotspots and emerging trends in specific fields within a specified period of time (18). CiteSpace produces a number of important metrics. The centrality based on structural hole theory, this measures how often a node lies on the shortest path between others. High-centrality nodes act as critical information hubs (21). The clustering method of CiteSpace is spectral clustering, which uses each vertex in the graph as a cluster and merges the clusters by calculating the similarity between different vertices (22). In the clustering analysis, cluster effectiveness is assessed by the modularity Q-score (network divisibility) and silhouette S-score (cluster homogeneity), with values approaching +1 indicating stronger clustering (23). In this Study, the cluster module value Q is set to 0.733 (Q>0.3), and the average contour value of the cluster S is set to 0.883 (S>0.5). This indicates that the clustering process is effective and reasonable. In the keyword clustering analysis results, “Cluster Size” denotes the number of keywords assigned to the cluster, indicating the scope and popularity of the research topic; “Silhouette Score” measures cohesion within the cluster and its separation from others (range: -1 to 1), where a value >0.7 (the empirical threshold) signifies strong, reliable cluster structure; “Mean Year” reflects the average year of first occurrence for keywords within the cluster, signifying the approximate time of the topic’s emergence or peak popularity; and the “Cluster Label (LLR)”, automatically extracted using the Log-Likelihood Ratio algorithm, represents the most thematically representative core keyword/phrase, serving as the identifier for the research theme. The burstness demonstrates a specific duration when a sudden change in the frequency of an element occurs, thus identifying emerging terms, which can represent trends and frontiers to some extent (24). CiteSpace visualizations represent elements (authors, countries, institutions, keywords) as nodes. Node size corresponds to publication count or frequency. Lines between nodes denote co-occurrence or co-citation, with thickness indicating relationship strength. Red nodes signify high activity (“hot”), and a purple outer ring indicates high centrality (>0.1) (15). With Citespace, the study group drew the cooperation map of the country, the co-cited reference and Keywords time zone diagram, and the references and keywords burst maps to realize the visualization analysis of research status, hotspots, and frontiers. Within the CiteSpace software, specific settings were configured: Time Slicing was set to 1999ingre with 1 year per slice. Various Node Types were offered, including Author, Country, Institution, Journal and Keywords. The Selection Criteria was established at Top N = 50, and Pruning options were chosen to include the “pathfinder”, “pruning of sliced networks”, and “pruning of merged networks”. Once these settings were finalized, the software was executed to produce a comprehensive visual map.

## 3 Results

### 3.1 Number of publications

**Figure 2:**
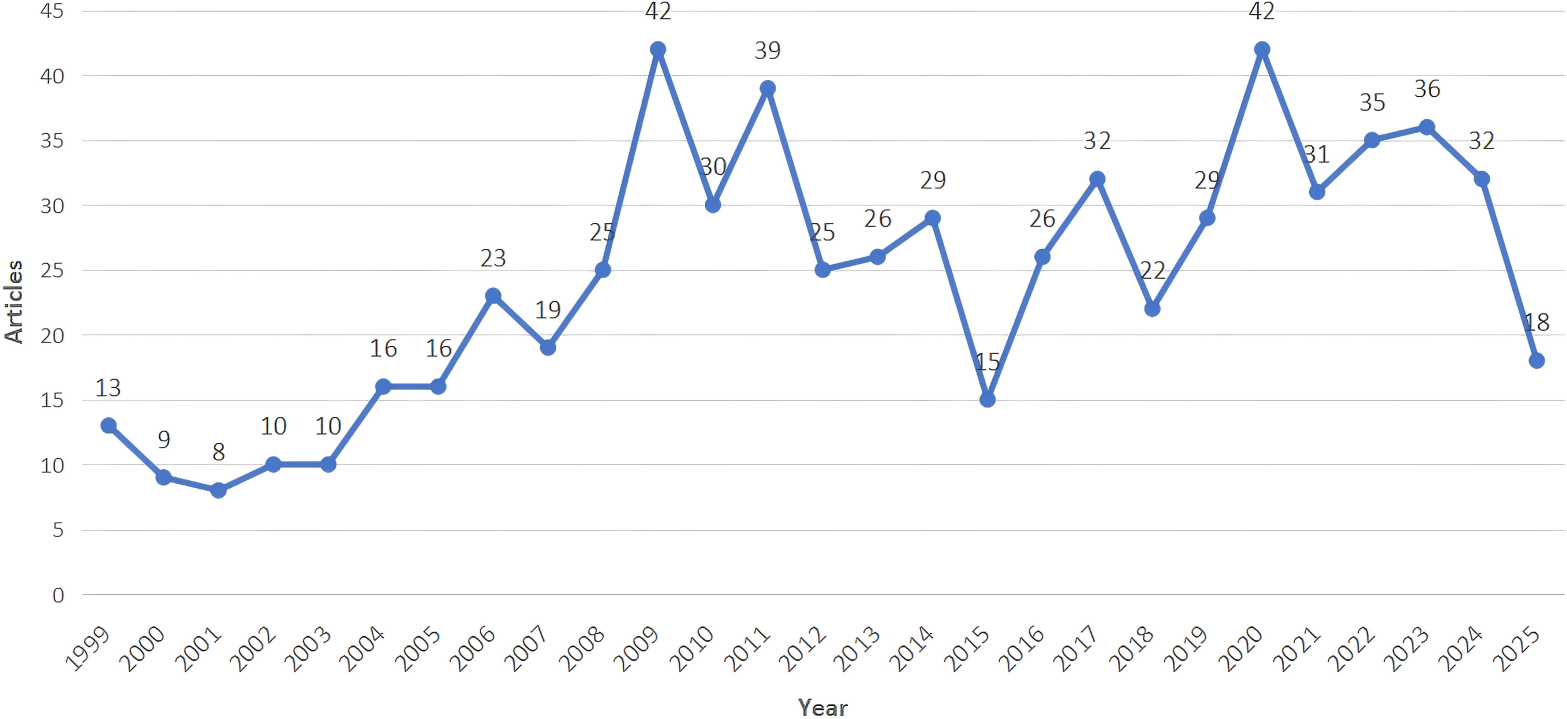
Number of articles published per year.

As illustrated in **Figure 2**, the trend of the number of publications in international studies on oncology genetic nursing has fluctuated in recent years, but overall, there has been an upward trend. Particularly, the analysis reveals that the period from 2001 to 2009 and 2015 to 2020 show the most significant growth in the number of published papers.

### 3.2 Analysis of countries/regions and institutions

**Figure 3:**
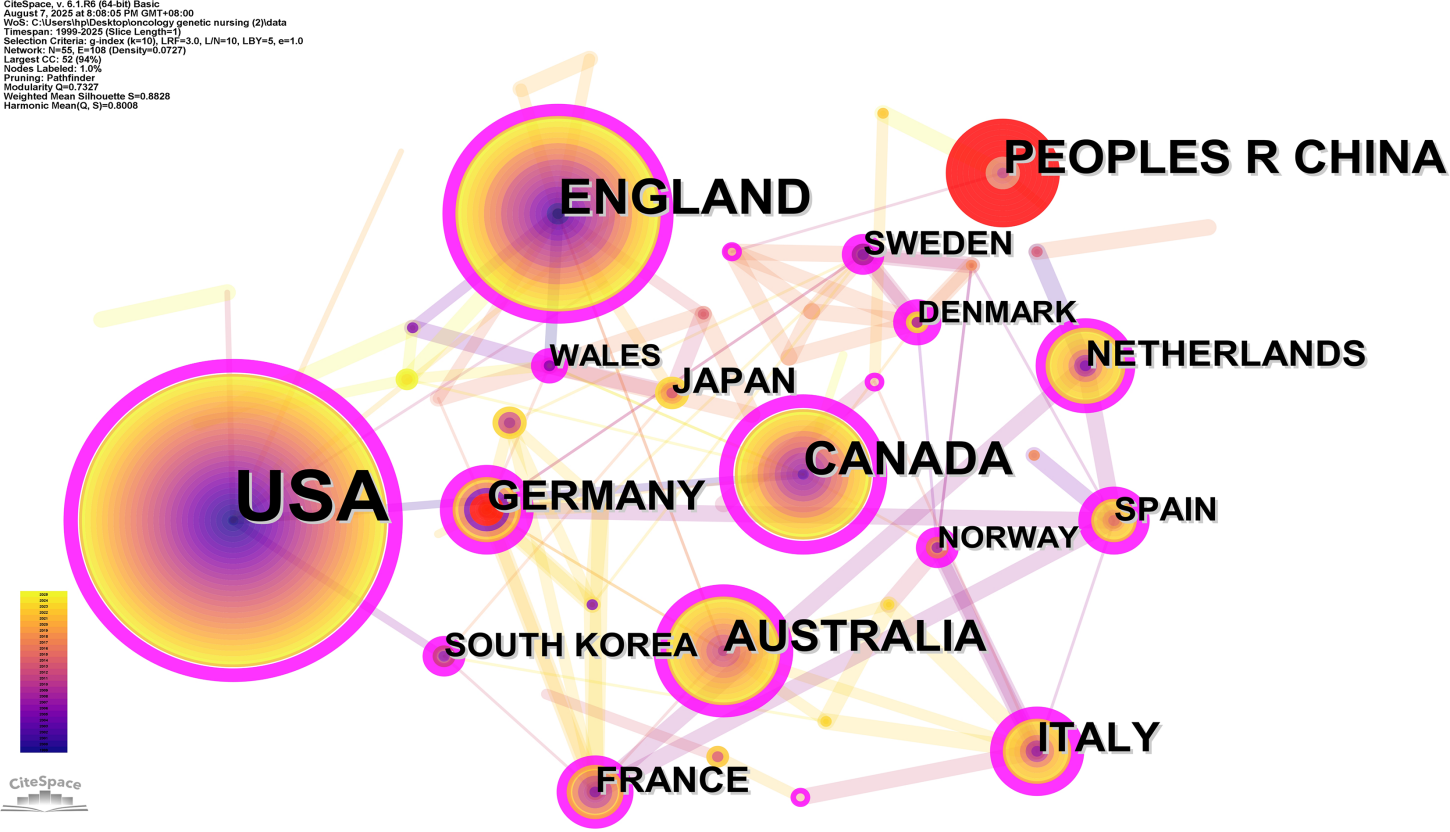
Visualization of research networks of country distribution on oncology genetic nursing.

A total of 54 countries participated in the studies analyzed, with 16 countries publishing more than 10 studies each. The United States (USA) emerged as the leading contributor, followed by the England and China. The top 10 contributing countries are detailed in **Table 2**. CiteSpace analysis was utilized to create a visualization of country collaborations; as depicted in **Figure 3**, the network consists of 55 nodes and 266 links, illustrating the interconnected academic collaborations among high-producing countries. The top five countries by centrality identified are Canada, the United States, Italy, Germany and England. Among these, Canada, the United States and Italy emerge as the top three countries in terms of centrality, with values of 0.49, 0.47, and 0.39. Analysis based on publication numbers and centrality metrics highlights Canada, the United States, Italy and Germany as the significant research powerhouses in this study.

**Table 2:** A list of the top 10 most country.

Rank	Country	Count	Centrality
1	USA	407	0.47
2	England	66	0.22
3	People’s Republic of China	49	0.10
4	Canada	47	0.49
5	Australia	41	0.20
6	Italy	27	0.39
7	Germany	26	0.37
8	Netherlands	20	0.14
9	France	16	0.20
10	Japan	16	0.03

USA, united States.

**Figure 4:**
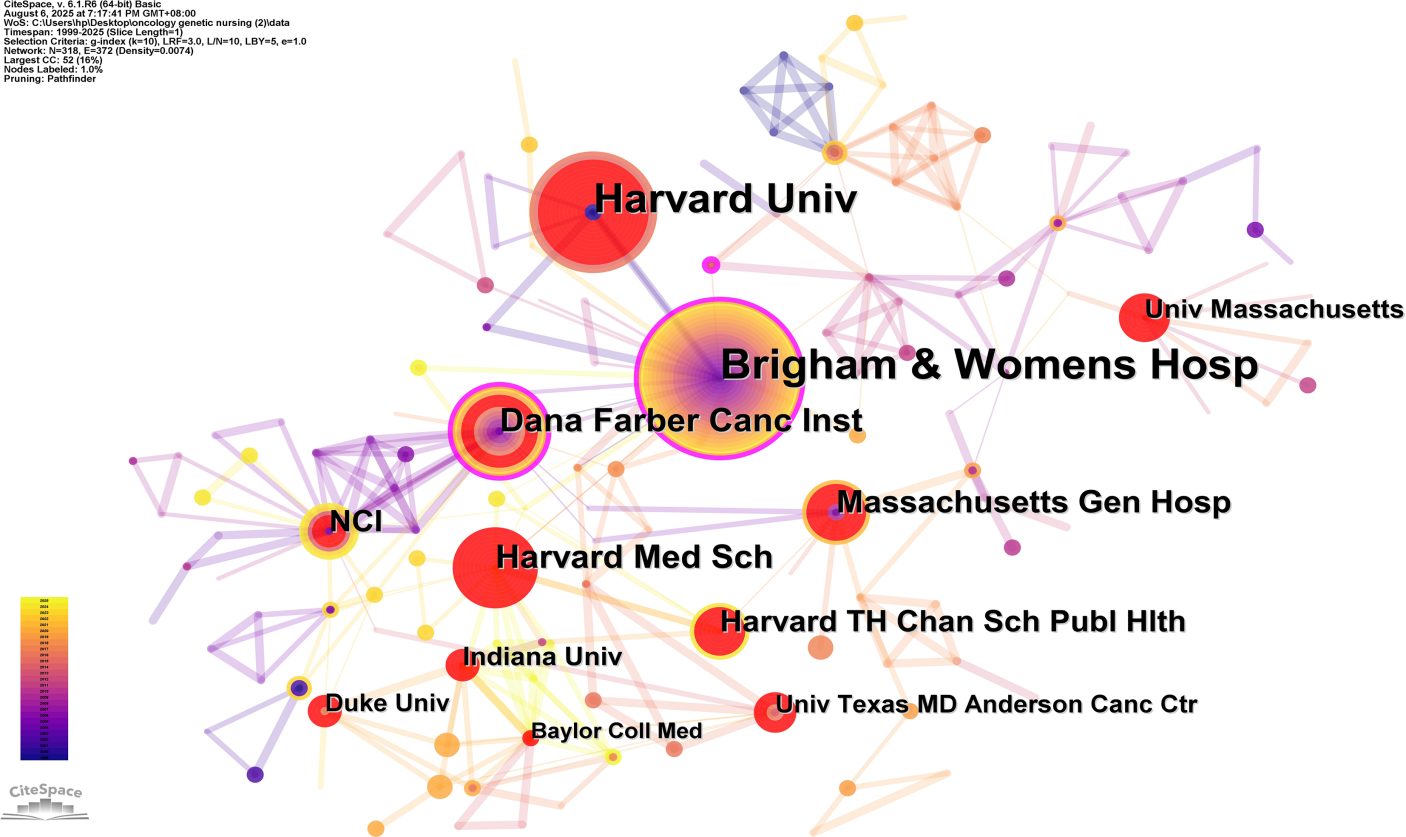
Visualization of research networks of institution distribution on oncology genetic nursing.

A total of 318 institutions participated in publishing research papers, with 49 institutions (15.41%) contributing more than 2 papers. The top 10 institutions, detailed in **Table 3**, each produced at least 14 papers. The Brigham & Women’s Hospital emerged as the leading contributor in this field, with a significantly higher number of publications compared to other institutions. Following the Brigham & Women’s Hospital, Harvard University and Dana Farber Cancer Institute demonstrated substantial contributions. Collaborative institution mapping results, illustrated in **Figure 4**, showcased 318 nodes and 461 links, indicating cooperative solid relationships. The top 10 institutions are all from the United States.

**Table 3:** A list of the top 10 most Institution.

Rank	Institution	Count	Centrality
1	Brigham & Women’s Hospital	114	0.17
2	Harvard University	102	0.01
3	Dana Farber Cancer Institute	38	0.10
4	Harvard Medical School	36	0.08
5	Massachusetts General Hospital	31	0.07
6	Harvard T.H. Chan School of Public Health	27	0.04
7	National Cancer Institute	25	0.07
8	Indiana University	15	0.00
9	University of Massachusetts	15	0.03
10	Duke University	14	0.01

### 3.3 Distribution of journals

**Figure 5:**
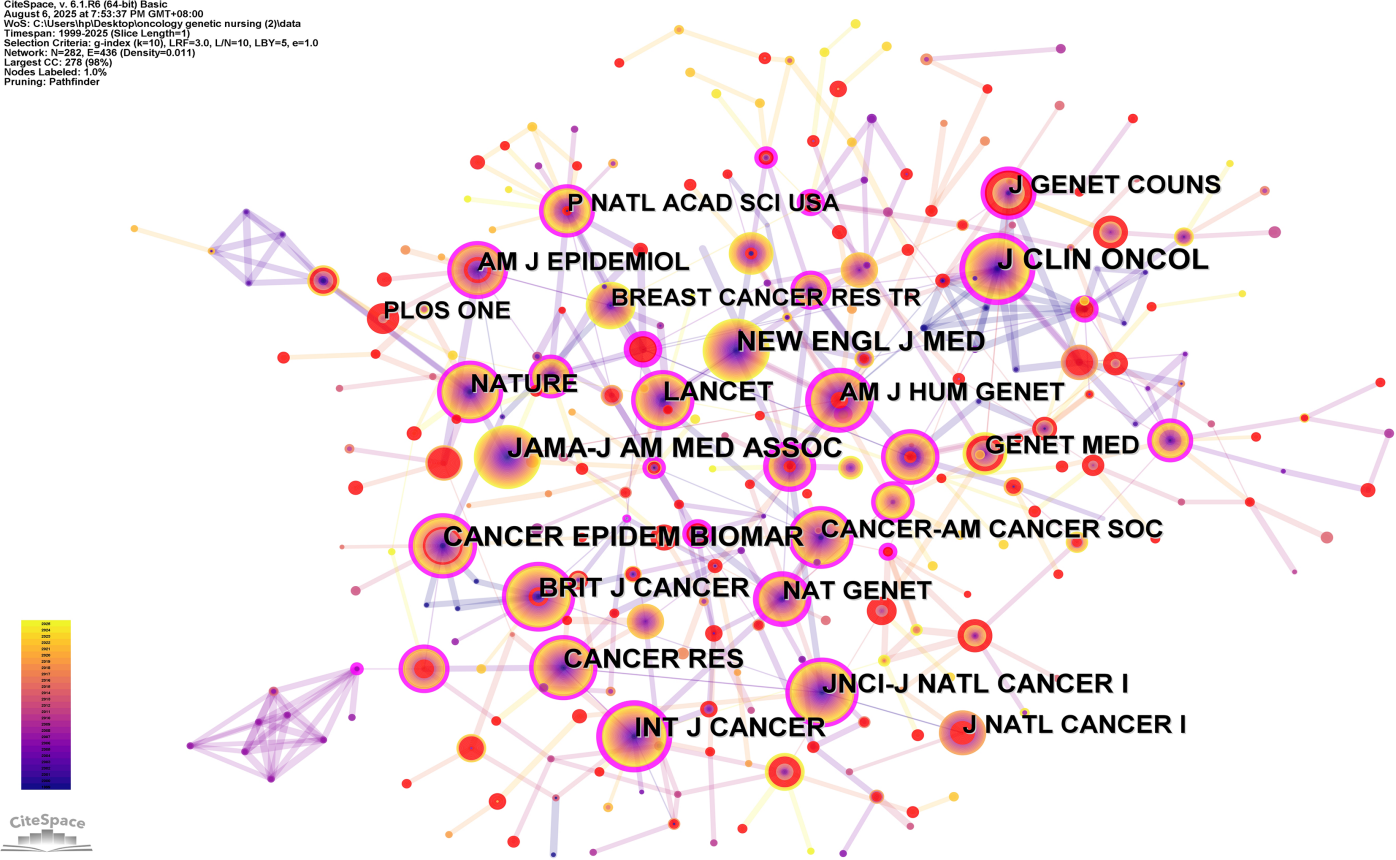
The co-citation journal analysis on Oncology Genetic Nursing.

A total of 282 academic journals have published articles on the research of oncology genetic nursing. **Table 4** displays the top 10 most cited journals. The impact of journals depends on the number of times they are collectively cited, which reflects their necessary influence on specific topics. The top five cited journals include Journal of Clinical Oncology (with 252 citations), Cancer Epidemiology Biomarkers&Prevention (with 222 citations), New England Journal of Medicine (with 222 citations), Jama-Journal of the American Medical Association (with 208 citations) and Cancer Research (with 182 citations) in **Figure 5**.

**Table 4:** A list of the top 10 most cited journals.

Rank	Cited journals	Citations	Centrality
1	Journal of Clinical Oncology	252	0.28
2	Cancer Epidemiology Biomarkers&Prevention	222	0.10
3	New England Journal of Medicine	222	0.11
4	Jama-Journal of the American Medical Association	208	0.06
5	Cancer Research	182	0.17
6	JNCI-Journal of the National Cancer Institute	173	0.15
7	International Journal of Cancer	166	0.24
8	British Journal of Cancer	159	0.13
9	Lancet	150	0.13
10	Natural Genetics	139	0.19

### 3.4 Analysis of authors

The papers were contributed by 337 authors. The top 10 authors in terms of number of publications are presented in **Table 5**. The top three most frequently cited authors were Colditz, Graham A (*N=47*), Hunter, David J (*N=32*), and Hallowell N (*N=26)*.

**Table 5:** A list of the top 10 most author and cited author.

Rank	Author	Count	Cited author	Citations
1	Hunter, David J	28	Colditz, Graham A	47
2	Kraft, Peter	27	Hunter, David J	32
3	De vivo, Immaculata	25	Hallowell N	26
4	Hankinson, Susan E	21	Calzone, Kathleen A	24
5	Han, Jiali	15	Hankinson, Susan E	23
6	Hankinson, Susan E	12	Han, Jiali	21
7	Chan, Andrew T	11	Rebbeck, Timothy R	20
8	Nan, Hongmei	9	Burke W	17
9	Colditz, Graham A	9	Haiman, Christopher A	16
10	Liu, Hongliang	8	Lerman C	15

**Figure 6:**
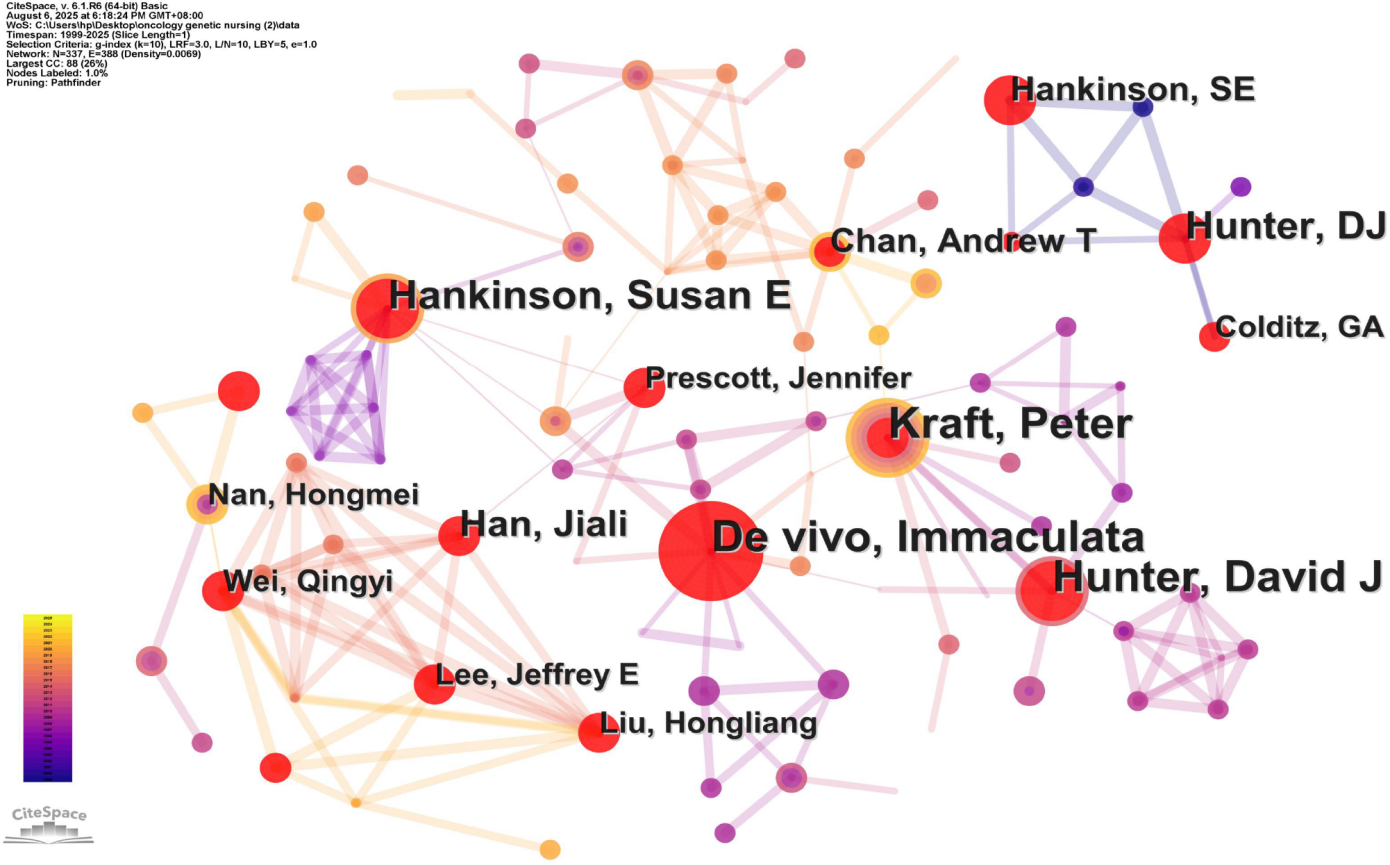
Author cooperative network analysis of oncology genetic nursing.

Co-cited authors refer to authors who are also cited in the article, and based on bibliometric analysis, a co-author network diagram is generated. According to the visualization analysis of CiteSpace co-cited authors, Colditz, Graham A ranked first among co-cited authors. As shown in **Figure 6**, this indicates the involvement of numerous research teams, including several highly productive authors contributing significantly to the field, such as Hankinson, Susan E, De vivo, Immaculata, Hunter, David J, and Kraft, Peter.

### 3.5 Keyword co-occurrence analysis

**Figure 7:**
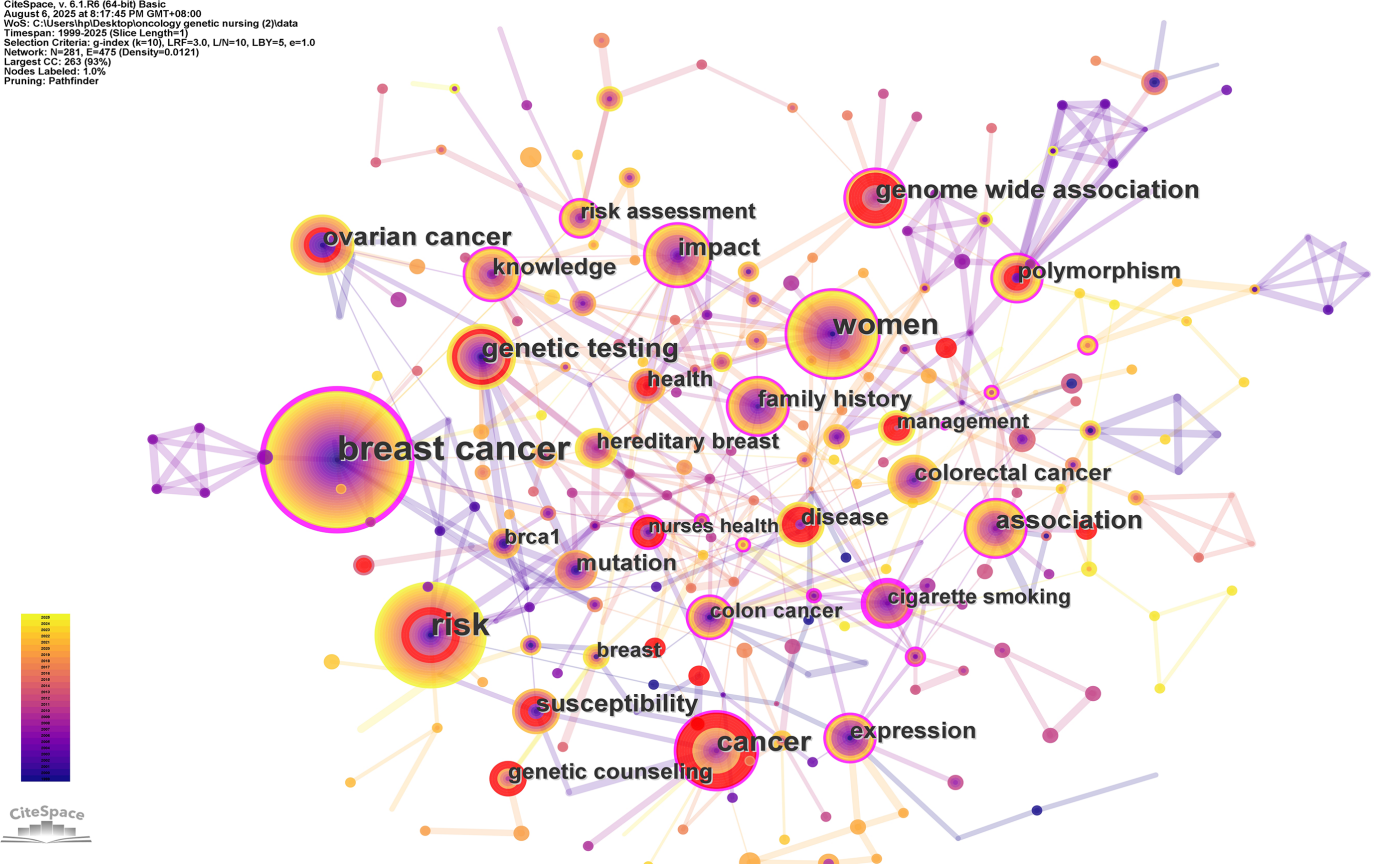
The network map of high-frequency keywords on oncology genetic nursing.

The top five high-frequency keywords in **Table 6** are “breast cancer” (n=146), “risk” (n=104), “women” (n=73), “genetic testing” (n=44), and”association” (n=39). Keywords with high intermediation are “cigarette smoking” (0.32), “breast cancer” (0.31), “family history” (0.19) and “polymorphism” (0.18) (**Figure 7**).In order to capture the key themes in studies related to oncology genetic nursing, keyword clustering was performed, and the top ten largest clusters were identified. (**Table 7**) They are as follows: “single-nucleotide polymorphism” (Cluster 0), “genetic testing” (Cluster 1), “radiation therapy” (Cluster 2), “single nucleotide polymorphisms” (Cluster 3), “health” (Cluster 4), “nurse practitioner” (Cluster 5), “risk assessment”(Cluster 6), “hereditary cancer syndromes” (Cluster 7), “body mass index” (Cluster 8), “nursing care” (Cluster 9). The largest thematic cluster (#0, 34 keywords), centered on single-nucleotide polymorphism (emerging in 2007), encompasses genetic testing and variation analysis, reflecting the foundational role of early-stage genetic research. Notably, Cluster #2 (Silhouette=0.99) demonstrates exceptional thematic coherence in cancer therapy and quality-of-life integration (2010), with keywords like “radiation therapy” and “quality of life” revealing the intrinsic link between clinical interventions and patient well-being. Emerging as a recent hotspot, Cluster #7 (2015) highlights innovations in hereditary cancer syndrome care, emphasizing the growing prominence of oncology nurses and clinical “mainstreaming” of genomics. Cross-domain convergence is evident in Cluster #3 (2012), where precision medicine penetrates primary care (“primary care”, “genomic testing”), and Cluster #6 (2010), where risk assessment drives personalized medicine (“risk prediction”, “hereditary syndromes”).

**Table 6:** Top 20 keywords in terms of frequency on oncology genetic nursing.

Rank	Keywords	Count	Rank	Keywords	Count
1	breast cancer	146	11	family history	27
2	risk	104	12	mutation	26
3	women	73	13	polymorphism	26
4	cancer	56	14	colorectal cancer	25
5	genetic testing	44	15	disease	24
6	ovarian cancer	40	16	expression	24
7	association	39	17	knowledge	24
8	genome wide association	35	18	genetic counseling	21
9	susceptibility	31	19	health	19
10	impact	28	20	risk assessment	19

**Table 7:** Clustering of keywords in the field of oncology genetic nursing.

Cluster ID	Size	Silhouette	Mean year	Label (LLR)
0	34	0.761	2007	single-nucleotide polymorphism (19.8, 1.0E-4); expression (14.98, 0.001); genetic variation (14.83, 0.001); genetic testing (9.91, 0.005); reproducibility (9.88, 0.005)
1	27	0.877	2008	genetic testing (25.38, 1.0E-4); mutation (11.65, 0.001); BRCA1 (8.53, 0.005); gastric cancer (7.76, 0.01); genetic counselling (7.13, 0.01)
2	21	0.99	2010	radiation therapy (8.33, 0.005); MTHFR (8.33, 0.005); quality of life (8.28, 0.005); BRCA2 (6.31, 0.05); breast cancer (5.62, 0.05)
3	20	0.933	2012	SNP (10.67, 0.005); precision medicine (9.59, 0.005); primary care (8.79, 0.005); genomic testing (7, 0.01); genetic counseling (6.17, 0.05)
4	19	0.884	2008	health (16.39, 1.0E-4); women (9.23, 0.005); pregnancy (8.18, 0.005); attitude (8.18, 0.005); disease (8.06, 0.005)
5	18	0.904	2012	nurse practitioner (14.35, 0.001); care (9.55, 0.005); health insurance (4.77, 0.05); outcomes (4.77, 0.05); superoxide dismutase (4.77, 0.05)
6	18	0.953	2010	risk assessment (20.62, 1.0E-4); risk prediction (11.67, 0.001); society (7.96, 0.005); hereditary breast and ovarian cancer syndrome (6.35, 0.05); personalized medicine (6.35, 0.05)
7	18	0.795	2015	hereditary cancer syndromes (10.97, 0.001); oncology nurses (7.92, 0.005); genomics (7.09, 0.01); mainstreaming (6.31, 0.05); young women (5.8, 0.05)
8	18	0.842	2008	BMI (8.21, 0.005); genetic susceptibility (6.58, 0.05); endogenous estrogen metabolite (5.95, 0.05); molecular epidemiology (5.95, 0.05); menstrual cycle (5.95, 0.05)
9	17	0.916	2012	nursing care (12.19, 0.001); parent (12.19, 0.001); decision making (7.43, 0.01); general anesthesia (6.08, 0.05); of life (6.08, 0.05)

LLR, Log-Likelihood Ratio; SNP, single-nucleotide polymorphism; MTHFR, methylenetetrahydrofolate reductase; BRCA1, Breast Cancer gene 1; BRCA2, BReast Cancer gene 2; BMI, body mass index.

### 3.6 Keywords with citation bursts

**Figure 8:**
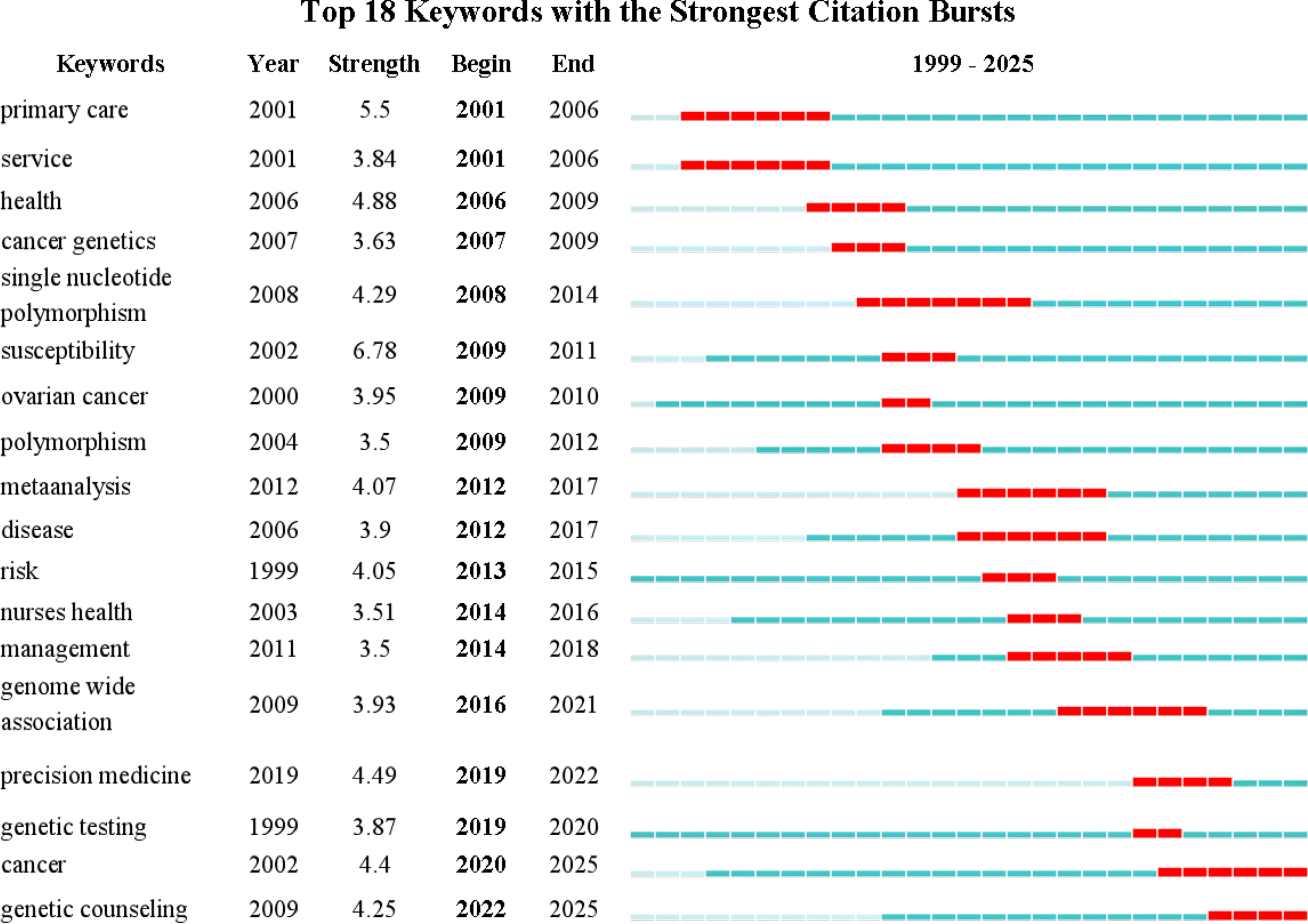
Top18 keywords with the strongest citation bursts.

Through the burst keyword analysis (**Figure 8**), there are a total of nine burst keywords. Combined with the keyword frequency, centrality and suddenness, it can be seen that the research hotspot of oncology genetic nursing has shifted from the initial primary care, health, susceptibility, polymorphism, etc. to meta-analysis, risk, management, Genome Wide Association, Genetic Testing, Precision Medicine continues to this day.

## 4 Discussion

### 4.1 General information

This study analyzed 658 oncology genetic nursing-related papers included in the Web of Science core collection, covering the period from 1999 to 2025, contributed by 337 scholars across 282 journals. CiteSpace analysis revealed a steady growth trend in academic output over the 26-year period, with the number of papers in 2020 reaching nearly five times the 2000 record, demonstrating the sustained vitality of academic research in this field.

The United States had the highest volume of oncology genetic nursing publications, surpassing England, the second highest-ranking country by over six times. The top 10 institutions in the field of oncology genetic nursing are all the United States, underlining its significant contributions to this field. While the United States accounts for 44.28% of total publications in the field of oncology genetic nursing research, network analysis shows that Canada and Italy are key knowledge intermediaries with significantly higher central values (0.46) than the United States. This suggests that these countries serve as primary bridges for facilitating the dissemination of oncogenetic nursing-related knowledge. The rapid development of oncology genetic nursing primarily from two factors: the establishment of a robust competency framework through systematic integration of genomics education (into curricula, licensing exams, and continuing education) (25, 26) and standardized professional certification (e.g., ANCC., genetic nurse credentialing) (27); Meanwhile, strong advocacy by professional organizations (such as the Oncology Nursing Society) and cross-national collaboration within Europe and North America created a rapid development in this field.

Among the top ten journals publishing in oncology genetic nursing, five are based in the United States and four in the United Kingdom, aligning with these nations’ dominant scholarly output in the field. The most frequently cited journal, Journal of Clinical Oncology (Oncology, JCR Q1), maintains a 2024 Journal Impact Factor of 41.9. Continuous tracking of research developments in these high-impact publications and their contributing authors provides critical insight into this rapidly evolving discipline. There are no nursing journals among the top 10 journals in terms of citation frequency. At the same time, highly cited authors and institutions are not from the nursing field. This is because oncology genetic nursing is still in its infancy in the nursing field, and the research foundation consists mainly of clinically related articles, resulting in fewer citations of nursing journals. This indicates that the field of oncology nursing is still in its early stages of development.

### 4.2 Knowledge base

From a historical perspective, genetic nursing in oncology emerged alongside the Human Genome Project (1990–2003). The first genetic nursing network in the United States was established in 1984, and the founding of the International Society of Genetic Nurses in 1988 marked a pivotal moment. Following the establishment of genetic nursing competency standards in Europe and the United States after the year 2000, the American Nurses Credentialing Centre began unified certification in 2014. Advanced Practice Genetic Nurses (APGNs) have gradually begun to operate outpatient clinics independently, particularly in the fields of oncology and obstetrics/gynecology. The core function of genetic outpatient clinics is to assess, manage, and prevent the risks of genetic diseases. The International Society of Nurses in Genetics (ISONG) defines genetic nurses as registered nurses who have received professional genetic knowledge training and education and are capable of providing genetic risk assessment, analysis results, and genetic counselling to patients with genetic risks or their affected blood relatives (28). Their primary roles and responsibilities include: assisting in the collection, recording, and interpretation of genetic information; assessing genetic risks; providing genetic information and resources to patients and counselees; participating in the development of risk management strategies for patients and families, and conducting follow-up and monitoring (28). Genetic services led by oncology genetic nurses are cost-effective and effective. Genetic counselling clinics led by oncology genetic nurses play an important role in primary care. A study on comprehensive cancer genetic care provided by genetic advanced practice nurses (27) examined the distribution of work time across different activities. The results indicated that cancer genetic nurses spent approximately 41% of their time on direct clinical care for patients and their families, including initial genetic counselling, telephone consultations, and follow-ups; the remaining time was allocated to indirect care activities, such as pre-consultation preparation, risk calculation, clinical trial registration, communication, education, and administration. Cancer nurses possess unique advantages in minimizing and managing the risks of hereditary cancers, thereby improving patient outcomes (29). A study on the feasibility and acceptability of genetic counselling clinics led by genetic nurses in primary care in the UK (9) found that patients reported high overall satisfaction, were satisfied with the time and distance required to travel to the hospital, and found the cost of visits to be low. With the sharp increase in demand for oncology genetic counselling, there is a shortage of genetic services professionals. Advanced practice oncology nurses will play a key role in cancer risk assessment, genetic counselling, genetic testing, management of hereditary tumour families, and psychosocial support. Although countries are continuously advancing the comprehensive development of genetic counselling and striving to professionalize it, currently, there remain issues such as insufficient theoretical knowledge of genetics among oncology nursing staff, inadequate mastery of specialized skills in cancer genetic counselling, the absence of a training system and certification for genetic specialty nurses, and deficiencies in genetic communication skills (30, 31). Therefore, nursing staff should actively participate in genetic counselling for cancer patients, continuously enhance their genetic knowledge and practical skills, strengthen their foundational knowledge of genetics and genomics, and conduct professional nursing work under scientific guidance to promote the development of specialized cancer nursing.

### 4.3 Hotspots and frontiers

From the keywords “breast cancer,” “ovarian cancer,” “women,” and “colorectal cancer”, it is evident that breast cancer was an initial research hotspot. Breast cancer patients are among the first to receive genetic evaluation (32). Over time, although “breast cancer” did not reappear at the forefront, it remained a continuously focused area of study (33). Meanwhile, research related to ovarian cancer, colorectal cancer also began to emerge (34, 35). This is related to the fact that these cancers are genetically correlated. The keywords “risk”, “susceptibility” and Cluster 8# risk assessment indicate that researchers are deeply concerned about the risk factors and genetic-related risk assessments for cancer. Risk factor analysis and disease risk assessment remain key research priorities in oncology genetic nursing. Researchers conducted in-depth explorations into the mechanisms of cancer development and discovered that various factors, including genetics, environment, and lifestyle, could influence its occurrence. In some developed countries in Europe and North America, extensive research has been conducted on risk assessment for genetically related cancers (36, 37). Risk factor analysis and disease risk assessment remain key research priorities in oncology genetic nursing. Mayo Clinic nurses conducted a study (38) that provided genetic mutation screening and interpretation of test results for a population, followed by statistical analysis of genetic data. The findings revealed that over half of participants (53.8%) carried at least one recessive disease-causing gene, while 44.5% exhibited genetic variants associated with multifactorial diseases. The keyword “genetic testing”, “genome-wide association”, “polymorphisms” along with Cluster 0# and 3# single-nucleotide polymorphism, Cluster 1# genetic testing focusing on the same, suggests that researchers at that time had started to pay attention to utilizing genetic testing as a means to predict and diagnose cancer. Performing genetic testing in high-risk populations for cancer has significant guiding significance for early cancer prevention. Nursing professionals play a vital role in facilitating informed decisions regarding genetic testing and/or treatment, as well as supporting patients’ choices for gene therapy. They assist patients in determining the necessity of genetic testing and selecting appropriate tests to identify potential genetic mutations within families, assess cancer risks for patients and other family members, calculate cancer risk for individuals with mutated genes, and provide counseling before and after genetic testing (39, 40). Study showed (8) that the average waiting time for results from a test was reduced to 35.8 days under a nurse-led genetic testing service. The keyword “impact” reflects that the influence of cancer on patients’ quality of life and socio-economic aspects is gradually being recognized. The keyword “knowledge” indicates that health education regarding cancer genetics is also a research hotspot. Studies have shown that inadequate understanding of cancer genetics, lack of screening awareness, and fear of the results are significant factors hindering the popularization of early cancer detection (41, 42). Nurses can address these issues by providing genetic knowledge to patients, high-risk populations for cancer, and their families, developing systematic and standardized health education programs, conducting long-term psychosocial follow-up, formulating preventive measures, and improving treatment outcomes. The keyword “genetic counseling” suggests Genetic counseling is also one of the research hotspots. International research on genetic counseling extends beyond patient populations to include healthcare professionals and caregivers. A Johns Hopkins Hospital study reveals that nursing teams serve as crucial sources of genetic information (43) Patients and their families actively seek guidance from nurses regarding genetic data, which helps them better understand disease risks and appropriate treatment strategies. Currently, advanced practice oncology nurses in genetics have become vital members of the multidisciplinary team for oncology genetic counseling. Clusters 5# Nurse Practitioner and 9# Nursing Care indicate that the training and certification of senior practicing oncology genetic nurses remain key research priorities. Further improvements to specialized accreditation systems, policy support for standardized services, and strengthening nurse-led health education programs continue to be actively explored (44, 45).

Through an in-depth analysis of emerging terms and their temporal distribution patterns, we have gained insights into the evolving trends in tumor genetics research. During the early phase from 2001 to 2006, “primary care”, “health” and “services” emerged as key themes, reflecting the academic community’s and clinicians’ growing emphasis on the quality of healthcare services. This trend may be linked to the global push for improving healthcare quality at the time, particularly the strengthening of primary healthcare services. There was also a growing focus on the training of nurses specializing in oncology genetics (39). From 2006 to 2012, the frequent appearance of keywords such as “cancer genetics,” “single nucleotide polymorphism,” and “susceptibility” marked a shift in research focus toward broader health issues, focusing on the association between genetic polymorphisms and disease susceptibility (46). These studies not only deepened our understanding of the genetic basis of diseases but also paved the way for the development of personalized medicine. Scientists during this period were increasingly interested in the association between specific genetic variations (such as single nucleotide polymorphisms, or SNPs) and diseases (47), and employed meta-analysis methods to integrate results from different studies to draw more reliable conclusions. Between 2012 and 2016, the terms “nurses”, “health” and “management” emerged, marking a shift toward nurse-coordinated care models. From 2016 to 2025, “genome-wide association,” “precision medicine,” “genetic testing,” and “genetic counseling” emerged as key themes, indicating the onset of the precision medicine era. These studies not only focused on disease diagnosis and treatment but also emphasized the importance of developing personalized treatment plans based on individual patient differences (48, 49). How to promote the development of precision medicine in oncology nursing is currently a research frontier (11). In recent years (2019–2025), “genetic testing” and “genetic counseling” have once again become popular keywords, reflecting the ongoing importance of these fields. This also marks the current research hotspot and frontier in building a nurse-led personalized genetic counseling and genetic testing service framework (50, 51).

## 5 Strengths and limitations

This study utilized the Web of Science core database and leveraged the citespace software tools to provide a comprehensive analysis of the literature on oncology genetic nursing from various perspectives. However, there are certain limitations to our approach. Firstly, our study only represents the current state of research. Secondly, our search was limited to a single database, which may have excluded potentially valuable information, and our results were restricted by search language (only English-language literature was included). In light of these constraints, future research should aim to conduct more inclusive systematic reviews of this area, incorporating a wider range of databases and addressing language limitations, to provide a more comprehensive exploration of the field of oncology genetic nursing.

## 6 Conclusion

This scientific metrology study focuses on oncology genetic nursing, revealing research hotspots and trends since the database’s establishment. We conducted a systematic review of the most influential countries, authors, and journals in this field and performed a thorough analysis of the correlation between basic scientific knowledge and keyword research trends. Our findings suggest that breast, ovarian, and colorectal cancers are the primary subjects of research. Current research priorities include offering nurse-led genetic risk assessments, genetic testing, and genetic counseling services, as well as improving the training of senior oncology genetic practice nurses. Additionally, applying precision medicine, genetic testing, and genetic counseling to cancer treatment has become a key research direction and frontier area. We hope this study provides researchers with clearer insights into field trends and helps them identify potential collaborators and directions for future research.

## Data Availability

The raw data supporting the conclusions of this article will be made available by the authors, without undue reservation.
